# A survey of mindset theories of intelligence and medical error self-reporting among pediatric housestaff and faculty

**DOI:** 10.1186/s12909-016-0574-8

**Published:** 2016-02-11

**Authors:** Mithila Jegathesan, Yaffa M. Vitberg, Martin V. Pusic

**Affiliations:** Department of Pediatrics, Three Lower Counties Community Services, Salisbury, Maryland USA; Department of Pediatric Emergency Medicine, Columbia University Medical Center, New York, New York USA

**Keywords:** Medical error, Psychology, Psychological theory, Intelligence, Mindset, Graduate medical education, Medical education, Pediatrics, Cohort studies

## Abstract

**Background:**

Intelligence theory research has illustrated that people hold either “fixed” (intelligence is immutable) or “growth” (intelligence can be improved) mindsets and that these views may affect how people learn throughout their lifetime. Little is known about the mindsets of physicians, and how mindset may affect their lifetime learning and integration of feedback. Our objective was to determine if pediatric physicians are of the "fixed" or "growth" mindset and whether individual mindset affects perception of medical error reporting.

**Methods:**

We sent an anonymous electronic survey to pediatric residents and attending pediatricians at a tertiary care pediatric hospital. Respondents completed the “Theories of Intelligence Inventory” which classifies individuals on a 6-point scale ranging from 1 (Fixed Mindset) to 6 (Growth Mindset). Subsequent questions collected data on respondents’ recall of medical errors by self or others.

**Results:**

We received 176/349 responses (50 %). Participants were equally distributed between mindsets with 84 (49 %) classified as “fixed” and 86 (51 %) as “growth”. Residents, fellows and attendings did not differ in terms of mindset. Mindset did not correlate with the small number of reported medical errors.

**Conclusions:**

There is no dominant theory of intelligence (mindset) amongst pediatric physicians. The distribution is similar to that seen in the general population. Mindset did not correlate with error reports.

## Background

People have long debated whether human qualities, such as intelligence, can be altered or if they are largely fixed from the moment of birth and difficult to change. According to Carol Dweck’s Mindset Theory, the view that one adopts can profoundly affect their life trajectory and level of achievement [1].

The “fixed” mindset upholds the belief that one’s human qualities are unalterable despite efforts to change them. Dweck argues that the “fixed” mindset creates an urgency to prove oneself and that any failure encountered may be perceived as a direct measure of competence and self-worth. Dweck’s research has shown that those who hold a fixed theory of intelligence are more likely than “growth” theorists to react helplessly in the face of achievement setbacks [1–3]. They are not only more likely to make negative judgments about their intelligence, but also more likely to show “negative affect and debilitation” after failure [4].

People of the “growth” mindset in theories of intelligence are somewhat different. “Growers” believe that they can always substantially change their intelligence through effort and continuous integration of feedback” [1]. A person of the “growth” mindset is conscious of and willing to voluntarily receive ego-threats to their perception of their innate ability, so that they may continually re-evaluate their learning process and maximize their potential. “The passion for stretching yourself and sticking to it, even (or especially) when it’s not going well, is the hallmark of the growth mindset. This is the mindset that allows people to thrive during the most challenging times in their lives” [1]. Empirical research in children, adolescents and adults in certain professional groups, including auditors and accountants has shown that modifying feedback according to these principles can be beneficial [5–8]. However, there are few studies examining physicians specifically, and their variation according to mindset [4].

Intelligence theory may have special relevance in the education of physicians: the rigors of the physician selection process may selectively favor one mindset over the other. Fear of performing poorly in front of colleagues and patients, when stakes can be high and situations life threatening, may favor a mindset that seeks out self-improvement and feedback. Physician education has always been dependent upon continuous re-evaluation and feedback and whether people are of the “fixed” or “growth” mindset may be specifically important in shaping this training, in order to promote lifelong learning and practice-based performance [4, 9].

One specific way in which physicians can constantly re-evaluate their skills and their intellect is to receive and review feedback from medical errors [9–12]. Throughout their training, physicians may receive threats to their perception of their innate abilities and clinical reasoning, especially when making and/or admitting to medical errors. Many studies have focused on the rate of medical errors and how they affect patient safety [9–16]. However, the way in which medical error reporting intersects with mindset theory has not been studied. Papadakis et al., in a case–control study of 239 doctors subjected to disciplinary action by medical boards demonstrated a significantly diminished ability for self-improvement [17]. Those who are“fixed” may be more reluctant to seek out and/or report errors that they themselves made, as opposed to those of the “growth” mindset who in theory are more open to learning from feedback [19–21]. In a recent review, Teunnisen and Bok state “*Although research within medical education is starting to look into the role of practitioners as active seekers of feedback, this issue is still under-explored. The concept of self-theories may be instrumental in furthering understanding of this topic”* [4].

Mindset theory would predict that individual physicians of different mindsets might react differently to the making of and admitting to a medical error. This variability in mindset and medical error reporting could have educational implications in clinical practice, as a subsequent change in mindset via targeted training could be used to promote feedback in medical education and promote improvement in clinical practice.

Our objective was to investigate how pediatric post-graduate trainees and attending physicians varied in terms of mindset. Further, we postulated that if we were to find significant variability, those classified as “fixed” might differ in the rate at which they report medical errors compared with clinicians classified as “growth”.

## Methods

### Study design and IRB statement

We performed a cross-sectional survey of pediatric residents, fellows and attendings at Morgan Stanley Children’s Hospital of New York-Presbyterian from March 22^nd^, 2011 – April 15^th^, 2011. The study was approved by Columbia University’s Institutional Review Board. Completion of the questionnaire was accepted as consent to participate in our study.

### Study population

We obtained email addresses for pediatric residents, fellows and attendings from publicly available lists on the university and hospital websites. We distributed our survey to all first, second and third year Pediatric residents training in the ACGME accredited pediatrics residency program at Morgan Stanley Children’s Hospital of New York-Presbyterian, as well as all fellows and attending physicians holding privileges there in the Department of Pediatrics. The attending physician population consisted of both part and full time physicians, including subspecialists and generalists.

### Survey

The survey was divided into four sections: 1) Inclusion Criteria 2) Medical Error and Near Miss Reporting 3) Theories of Intelligence & Morality Statements 4) Demographics. Medical error questions were asked before the Theories of Intelligence questions; we did not describe the aims of the study to respondents. The mindset questions were four statements about basic intelligence, which have been previously validated by Dweck, et al. and used in subsequent studies by other authors: 1) “You have a certain amount of intelligence, and you can't really do much to change it.” 2) “Your intelligence is something about you that you can't change very much.” 3) “To be honest, you can't really change how intelligent you are.” 4) “You can learn new things, but you can't really change your basic intelligence” [3, 18]. The survey instrument was pilot tested for readability on 5 medical students and 5 residents and modified based on their feedback.

Mindset theory would predict that different mindsets would react differently to the ego-threat of making and admitting to a medical error. Those holding a “fixed” mindset might view the public reporting of an error as a direct threat to their self-concept and, therefore, might be more reluctant to report errors that they themselves had made, rather than “growth mindset”individuals who are thought to be more open to learning from feedback [19–22].

We defined an *actual medical error* as a preventable adverse event or omission that affected a patient by prolonging treatment or causing emotional or physical consequences, such as discomfort, disability or death and that would be judged as wrong by knowledgeable peers [10].

Respondents were asked to report how many actual medical errors they themselves had made and how many they had observed others make over the past 6 months. Perceived consequences to patients and feelings of responsibility for the actual medical error were also recorded. Respondents were also asked to volunteer information about their perceived ‘maximum’ error, the error they perceived to provoke the worst consequence.

### Procedure

We used Survey Monkey™ (surveymonkey.com) to distribute the questions electronically to our study population. Survey Monkey™ is a password protected web-based survey tool that tracks completed questionnaires, while hiding the link between the respondent and the questionnaire [23]. Using this option, there was no connection between the respondent and their answers, and anonymity was maintained. Completion of the questionnaire was accepted as consent to participate in our study.

Following the Dillman method, we sent reminder emails to non-respondents [24]. After recruitment was completed and all survey responses were collected, participants were awarded compensation in the form of a $5 gift card. The funds for these gift cards came from an internal education fund.

### Data analysis

An overall distribution of answers was tabulated among the respondents and analyzed using Carol Dweck’s formula for mindset scoring [2, 3]. The mindset theory items were reviewed and an individual mean theory of intelligence score calculated, with the low end (1) representing a “fixed” mindset, and the high end (6), representing a “growth” mindset. Individuals with a score from 3.1-3.9 were excluded as being equivocal, as per published guidelines [2, 3].

We conducted cross-tabulations relating the correlation between “fixed” and “growth” mindset and self-perception of actual medical errors made. In addition to descriptive statistics reporting the main results of the survey, we also conducted a number of secondary analyses. We verified comparisons statistically with one-way ANOVA testing, where the dependent variable was normally distributed, and Kruskal-Wallis testing, where it was not.

## Results

### Demographics

From March 22^nd^, 2011 to April 15^th^, 2011, we received 221/349 (63 %) responses, but only 176/349 (50 %) had answered all questions. 46/176 (26.2 %) were residents, 36/176 (20.4 %) were fellows and 94/176 (53.4 %) were attendings. 60 pediatric residents, 44 fellows and 245 attendings were emailed the survey, placing the resident response rate at 77 %, the fellow response rate at 82 % and the attending response rate at 38 % respectively. 57/176 (32.3 %) of respondents were male.

### Self-theories of intelligence

**Fig. 1:**
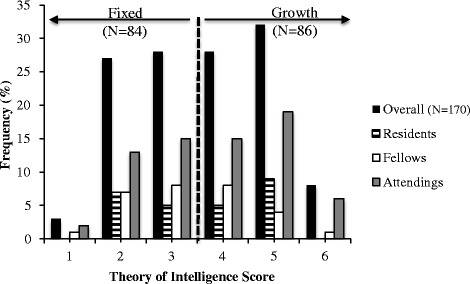
Frequency distribution of survey respondents by mindset score:  Overall (*N* = 170)  Residents  Fellows  Attendings *Graph separated into Fixed and Growth sections

Theory of intelligence score was calculated for 176 respondents and frequency of responses for the four intelligence theory items was further subdivided by resident, fellow and attending. Six individuals were excluded from the data analyses, as their scores were tabulated at 3.1-3.9, scores that are ‘indeterminate or equivocal’, per published guidelines [2, 3]. 84 (49.1 %) were classified as having a “fixed” mindset while 86 (50.9 %) were classified as having a “growth” mindset (Table 1, Fig. 1).

**Table 1:** Mindset measure and error reporting by level of training

	Resident	Fellow	Attending	Overall
	*N* = 46	*N* = 36	*N* = 94	*N* = 176
Mean theory of				
Intelligence score (SD):	3.66 (1.22)	3.40 (1.15)	3.66 (1.29)	3.64 (1.22)
Fixed (1–3)	20	19	45	84
Growth (4–6)	23	16	47	86
Indeterminate (3.1-3.9)	3	1	2	6
Median errors				
Reported (IQR) by:				
Self	1 (0, 2.75)	0 (0,1)	0 (0,0)	0 (0,1)
Others	3 (0,3)	3.5 (0,4)	1 (0,4)	2 (0,5)

^a^Neither Mindset measures nor Numbers of Errors reported differed significantly by Training Level

Of the 84 “fixed” respondents, 21 (25 %) were male compared with 35 (41 %) of the 86 “growth” respondents (*p* = NS). The proportion of physicians of each mindset type did not vary significantly by level of training: 20 (46.5 %) residents, 19 (54.3 %) fellows and 45 (48.9 %) attendings held the “fixed” mindset (Table 1).

### Medical error reporting

Overall, 41.9 % of 170 participants listed at least 1 self-reported medical error in the previous 6 months (58.1 % with 0 errors, 21.5 % with 1 error, 11.3 % with 2 errors and 9.1 % with 3 or more errors). A median of 0 self-reported errors (IQR 0,1; Maximum 15) was calculated over the previous 6 months vs. a median of 2 observed errors (IQR 0, 5; Maximum 30) over the same time period. This pattern did not differ by training level (Table 1).

### Relationship between medical error reporting and mindset

Whether reporting self-errors or those of others, there was no difference in the number of errors reported between “growth” and “fixed” individuals (Table 1). While residents, fellows and attendings may have different levels of knowledge to discern when an error occurs, these differences did not affect their report of self-errors. For example, most “maximum” errors described were related to dosing of medications and miscommunication, regardless of stage of training.

## Discussion

In this survey of pediatricians and pediatric housestaff, we hypothesized that there would be differences in the “fixed” vs. “growth” continuum in physicians, and found that clinicians could indeed be differentiated with regard to their individual theory of intelligence. Overall, this did not vary with training stage, with approximately half of each group representing each mindset. These proportions are comparable to results obtained by Dweck, et al. in studies done in adolescents as well as college students and, therefore, may be comparable to variations in mindset seen in the general population [2, 25, 26]. Previous studies have shown that roughly 20 % of individuals may fit partially into both groups, but most research has shown that individuals are equally divided into either mindset [26].

Differences in mindset may have educational implications. There is a body of research literature that shows that those with “growth” mindsets learn better. Dweck et al. followed pre-med students of the “fixed” mindset throughout their first semester of Chemistry in college. They were shown to have interest in a given assignment only when they did well right away and not when they were confronted with challenge or failure. In another study, adolescents of the “growth” mindset were shown to push themselves to increase their abilities and maximize their potential, even in the face of failure [2, 3]. Thus, in multiple settings, individuals with a “growth” mindset are more likely to respond to negative feedback with a renewed effort to achieve [25]. On the other hand, those subscribing to a “fixed” mindset may make inaccurate global self-judgments and assume a helpless response pattern in the face of adversity [18, 27, 28]. Doctors subject to disciplinary action by medical boards were strongly associated with demonstrating a diminished ability for self-improvement and response to feedback in medical school, as illustrated clearly by Papadakis et al [17]. For a physician committed to lifelong learning, feedback may contain useful information that can help correct future errors and promote achievement of mastery [4].

Educational interventions can beneficially change mindsets. Adolescents of the “fixed” mindset were taught the “growth” mindset and were able to significantly improve test scores despite negative outcomes or feedback, thereby maximizing their potential [2, 3]. Aronson, Fried and Good randomized 109 Stanford undergraduates into three groups: a control group, one where they promoted a “growth” mindset and one where they promoted a “fixed” mindset [29]. After the intervention, the growth group showed greater enjoyment of academics, greater identification with learning and a higher subsequent Grade Point Average [29]. Several studies have shown that students with a “growth” mindset tend to use deeper learning strategies and engage in active self-regulation of their motivations and emotions to improve practice-based performance [3, 18, 22, 26].

Our study participants have all successfully negotiated a rigorous academic gauntlet that required competitive SAT scores, MCAT scores and GPAs throughout their academic careers. If mindsets are stable within people over time, we would predict that those holding a “fixed” mindset may be under-represented in this high-achieving group. Instead, our community of pediatricians shows the same fixed-growth ratio as quoted in previous reports of children, college students and accountants [2, 5–8, 18, 25].

The question remains whether the “fixed” physicians have not been able to achieve as much as their “growth” counterparts due to their mindset, or whether there are actual compensatory benefits to having a “fixed” mindset that allow one to achieve just as well. Qualities of each mindset may be used and tailored to specific situations, to maximize achievement and improve clinical practice [19, 27, 28, 30, 31].

We speculated that if we found differences in mindset, an important area where these differences might have an impact, is in the reporting of medical errors. If a growth mindset leads to greater resilience and positive change in the face of negative feedback, then for physicians who have made an error, having a “growth” mindset may be beneficial [19, 27, 30, 31]. Conversely, those physicians with a “fixed” mindset might find the effect of an error deleterious [20–22, 30, 31]. Theories of intelligence may influence the outcomes of how any situation is perceived, which emotions are provoked as a result of that situation, and what action a physician may ultimately bring to the next situation [4]. However, in our simple survey, we did not find a correlation of mindset with the rate of reporting of medical errors. This finding is far from conclusive. Future research may still be able to delve further into evaluating whether variability in mindset affects a clinician’s response to error or other feedback.

Our study had several limitations. The response rate for the trainees was >75 % but for attendings it was only 38 % leaving those results open to response bias. Self-reporting of medical errors may be an underestimate since reporting may be biased for fear of social stigma, despite our assurances of confidentiality and anonymous survey methodology. Our measure of self-reported error, derived from previous research, demonstrated relatively little variability, potentially biasing our results to not finding a difference despite the sample size of 176. Finally, our study was carried out at a single institution and therefore the results may not necessarily be applicable to other institutions or contexts.

Future studies can elucidate how mindset interacts with a physician’s response, when a physician is confronted with threats to self-perception of intelligence and worth and whether these responses have implications for future clinical decision-making and practice-based performance in medicine.

## Conclusions

Theories of intelligence can range along a spectrum from “fixed” to “growth”. In this study, we show that pediatric trainees and practicing pediatricians show variation in mindset. These differences may have educational implications as mindset training and exploration of the benefits of each mindset can be used to maximize potential and promote lifelong learning with integration of feedback.
